# Consensus clustering and novel risk score model construction based on m6A methylation regulators to evaluate the prognosis and tumor immune microenvironment of early-stage lung adenocarcinoma

**DOI:** 10.18632/aging.206004

**Published:** 2024-07-05

**Authors:** Miao He, Yuxue Zhi, Chao Li, Changming Zhao, Guangquan Yang, Jing Lv, Hong You, Hai Huang, Xiaoyu Cao

**Affiliations:** 1 Department of Radiation Oncology, People’s Hospital of Deyang, Deyang 618000, Sichuan, P.R. China; 2 Department of Cardiovascular Surgery, People’s Hospital of Deyang, Deyang 618000, Sichuan, P.R. China

**Keywords:** early-stage lung adenocarcinoma, tumor immune microenvironment, m^6^A methylation regulators, risk score, prognostic model

## Abstract

Background: The aim of this study was to investigate the correlation between m^6^A methylation regulators and cell infiltration characteristics in tumor immune microenvironment (TIME), so as to help understand the immune mechanism of early-stage lung adenocarcinoma (LUAD).

Methods: The expression and consensus cluster analyses of m^6^A methylation regulators in early-stage LUAD were performed. The clinicopathological features, immune cell infiltration, survival and functional enrichment in different subtypes were analyzed. We also constructed a prognostic model. Clinical tissue samples were used to validate the expression of model genes through real-time polymerase chain reaction (RT-PCR). In addition, cell scratch assay and Transwell assay were also performed.

Results: Expression of m^6^A methylation regulators was abnormal in early-stage LUAD. According to the consensus clustering of m^6^A methylation regulators, patients with early-stage LUAD were divided into two subtypes. Two subtypes showed different infiltration levels of immune cell and survival time. A prognostic model consisting of HNRNPC, IGF2BP1 and IGF2BP3 could be used to predict the survival of early-stage LUAD. RT-PCR results showed that HNRNPC, IGF2BP1 and IGF2BP3 were significantly up-regulated in early-stage LUAD tissues. The results of cell scratch assay and Transwell assay showed that overexpression of HNRNPC promotes the migration and invasion of NCI-H1299 cells, while knockdown HNRNPC inhibits the migration and invasion of NCI-H1299 cells.

Conclusions: This work reveals that m^6^A methylation regulators may be potential biomarkers for prognosis in patients with early-stage LUAD. Our prognostic model may be of great value in predicting the prognosis of early-stage LUAD.

## INTRODUCTION

Non-small-cell lung cancer (NSCLC) accounts for 85% of cases of lung cancer [1]. Among NSCLC, lung adenocarcinoma (LUAD) is the most common [2]. One of the deadliest and most aggressive tumor kinds, LUAD has a less than 5-year overall survival [3]. Therefore, early diagnosis and treatment of LUAD are particularly important. Low-dose computed tomography (CT) can be helpful in the diagnosis of early-stage LUAD [4]. Surgical resection and radiotherapy are commonly used in early-stage LUAD [5]. However, overall survival after treatment is not ideal [6]. Recently, immunotherapy has become a new type of treatment. This method provides new ideas for clinical treatment management. The changes of immune microenvironment of early-stage LUAD affect disease progression [7, 8]. Therefore, in order to accurately predict the prognosis and significantly optimize immunotherapy management of early-stage LUAD, further exploration of the regulatory mechanisms of tumor immune microenvironment (TIME) is needed.

The modification of RNA by m^6^A is determined by the dynamic interaction between its methyltransferase (writer), binding protein (reader) and demethylase (erase) [9]. It has recently been discovered that m^6^A methylation regulators are crucial for TIME. The ALKBH5 can improve the efficacy of immunotherapy by regulating lactate content and inhibiting immune cell accumulation in TIME [10]. Evaluation of m^6^A modification patterns in patients with gastric cancer can improve our understanding of the characteristics of TIME infiltration and guide immunotherapy strategies more effectively [11]. The METTL3- or METTL14-deficient increases cytotoxic tumor-infiltrating CD8 T cells and alters TIME [12]. Therefore, exploring the role of m^6^A methylation regulators in TIME may be helpful for the implementation of immunotherapy.

According to earlier research, m^6^A methylation regulators are essential for controlling lung cancer. YTHDC2, a m^6^A reader inhibits the development of LUAD by SLC7A11-dependent antioxidant function. The expression of m^6^A methyltransferase METTL3 is increased in LUAD, and can regulate the growth of cancer cells [13]. The m^6^A methylation regulators also regulate the development of LUAD by regulating the TIME [14, 15]. Nonetheless, the potential mechanism of m^6^A methylation regulators in immune infiltration in the TIME of early-stage LUAD remains unclear.

Herein, all data of early-stage LUAD were downloaded from public databases. Then, the expression and consensus cluster analyses of m^6^A methylation regulators in early-stage LUAD was performed. Two subtypes of early-stage LUAD were identified. Subsequently, we also constructed a prognostic model.

## MATERIALS AND METHODS

### Data sources

All data of LUAD were obtained from TCGA (https://tcga-data.nci.nih.gov/tcga/) and GEO (http://www.ncbi.nlm.nih.gov/geo) databases. Early-stage LUAD patients with TNM stage I-II and survival information were selected. UCSC Xena (https://gdc.xenahubs.net) was used to download RNA sequencing data and clinical information in TCGA. The original “CEL” file of the GSE31210 dataset was downloaded from GEO database. Finally, 398 early-stage LUAD samples and 59 paracancerous samples were obtained from TCGA database, and 226 early-stage LUAD samples and 20 paracancerous samples were obtained from GEO database (Supplementary Table 1).

### Expression of m^6^A methylation regulators

Herein, 22 m^6^A methylation regulators were collected from the published literature [9, 16, 17]. These 22 m^6^A methylation regulators include 2 erasers (ALKBH5 and FTO), 12 readers (IGF2BP1, IGF2BP2, IGF2BP3, LRPPRC, YTHDC1, YTHDC2, YTHDF1, YTHDF2, YTHDF3, FMR1, HNRNPA2B1 and HNRNPC) and 8 writers (KIAA1429, METTL3, RBM15, RBM15B, WTAP, ZC3H13, METTL14 and METTL16). Then, we investigated the differential expression of these molecules in early-stage LUAD and normal tissue in TCGA. We also analyzed somatic mutations of 22 m^6^A methylation regulators in early-stage LUAD. Mutation data were obtained from TCGA.

### Unsupervised clustering

The “ConsensusClusterPlus” software [18] (http://www.bioconductor.org/) was used to divide patients with early-stage LUAD into different clusters. Then, the expression of 22 m^6^A regulators in different clusters was analyzed. Subsequently, we analyzed clinicopathological features and survival among early-stage LUAD patients in different clusters. Principal component analysis (PCA) was used to evaluate gene expression patterns between different clusters.

### Immune cell infiltration in TIME

We obtained gene set from Charoentong’s study that marked each TIME infiltration immune cell type [11, 19]. The enrichment score calculated by single-sample gene-set enrichment analysis (ssGSEA) was applied to represent the relative abundance of each TIME infiltration immune cell in each sample [20, 21]. The R “ESTIMATE” package was used to obtain the immune score, tumor purity and ESTIMATE score of patients [22]. The expression of PD-L1 in clusterA and cluster was tested. The R “GSVA” package was used for gene set variation analysis (GSVA) [23].

### Prognostic model

The correlation between each m^6^A regulator and the prognosis of patients with early-stage LUAD was examined using univariate analysis (P<0.05). Least absolute shrinkage and selection operator (LASSO)-Cox regression analysis was applied to determine the prognostic model. The risk score of each sample is calculated as follows: $Risk\;Score=\sum_{i=1}^{n}\left(\exp_{i}*\;\beta_{i}\right)$. Patients were categorized into high and low risk groups based on the median risk score. Subsequently, Kaplan-Meier (KM) analysis was carried out. The prognostic model’s accuracy was evaluated using receiver operator characteristic (ROC) analysis. The area under the curve (AUC) is an evaluation index of model performance. The AUC ranges from 0 to 1, where 0.6-0.7 indicates sufficient diagnostic accuracy [24]. Moreover, we also used univariate and multivariate Cox regression analysis to determine whether risk score was an independent prognostic factor.

### Risk score and immune cells

To research the correlation between the risk score and immune cell infiltration, we examined the relationship between the risk score and the infiltration level of 6 immune cells based on TIMER (https://cistrome.shinyapps.io/timer/). We also used the TIMER to evaluate the impact of somatic cell copy number alternations (CNAs) of m^6^A methylation regulators on the infiltration level of immune cell. The GISTIC 2.0 database was utilized in the TIMER.

### Validation of model genes expression in clinical tissue samples

Totally, 13 patients were included in this study, and the specific clinical information is displayed in Supplementary Table 2. Early-stage LUAD tissue and adjacent normal tissue were obtained for real-time polymerase chain reaction (RT-PCR) validation. Total RNA of samples was extracted using TRIzol kit. Subsequently, the FastKing cDNA first strand synthesis kit (TIANGEN, KR116) was used for reverse transcription. Finally, SuperReal PreMix Plus (SYBR Green) (TIANGEN, FP205) was used for RT-PCR validation. ACTB was used as internal reference. The data were calculated by 2^-ΔΔCt^ method [25].

### Validation of *in vitro* cell experiments

Cell line NCI-H1299 (Procell, CL-0165) was used for cell validation. The model gene HNRNPC was selected for knockdown and overexpression in NCI-H1299 cells. Overexpression lentivirus (vector: GV492) and target gene RNA interference lentivirus (vector: GV493) were purchased from Shanghai Jikai Gene Technology Co. The effect of HNRNPC on the migration of NCI-H1299 cells was analyzed by cell scratch assay. The cell density of knockdown control group, knockdown group, overexpression control group and overexpression group was adjusted to 2×10^5^ cells/well and seeded in 6-well plate, with 3 replicates in each group, and incubated at 37° C, 5% CO_2_. After the cells had grown to a monolayer, scratches were made with the micro pipette tip, and washed gently twice with phosphate buffer saline (PBS) to remove the scratched cells. After that, the culture was continued, and samples were taken and photographed according to the culture time of 0h, 24h and 48h. In addition, the effect of HNRNPC on the migration and invasion of NCI-H1299 cells was detected by Transwell assay. For Transwell assay, the cell density was adjusted to 4×10^4^ cells/well and seeded in 24-well plates. Cells on the bottom surface of the Transwell chamber were fixed with precooled ethanol for 30 min. Crystal violet solution was stained for 30 min and observed under a microscope.

### Statistical analysis

Wilcoxon test was applied to statistically analyze the differential expression of genes and the infiltration level of immune cell. The log-rank test was used to analyze significant differences in survival between different groups in Kaplan-Meier. In RT-PCR, the data were statistically analyzed by T-test. R software (version 3.6.3) was used for all statistics.

### Availability of data and materials

We searched for LUAD public gene expression data and complete clinical annotations from GEO (http://www.ncbi.nlm.nih.gov/geo) and TCGA (https://tcga-data.nci.nih.gov/tcga/) databases. The accession numbers are GEO: GSE31210 (Platform: Affymetrix Human Genome U133 Plus 2.0 Array) and TCGA: LUAD (Platform: Illumina RNAseq), respectively.

## RESULTS

### Genetic alterations of m^6^A methylation regulators in patients with early-stage LUAD

**Figure 1:**
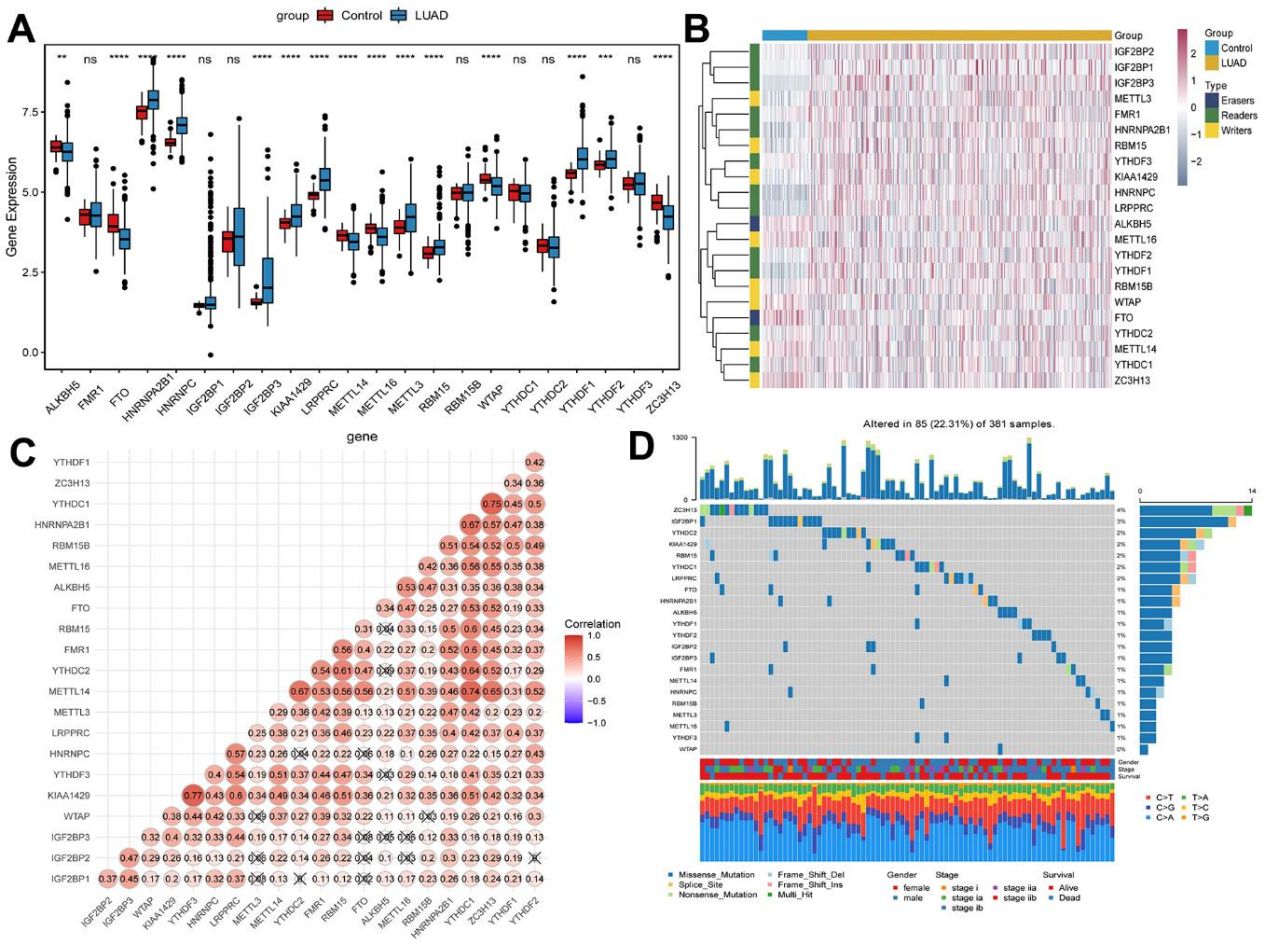
**Expression of m^6^A methylation regulators in early-stage LUAD.** (**A**) Expression boxplot of m^6^A methylation regulators in early-stage LUAD tissues and normal control tissues. *, P < 0.05; **, P < 0.01; ***, P < 0.001; ****, P < 0.0001. (**B**) Heatmap of m^6^A methylation regulators expression in early-stage LUAD tissues and normal control tissues. Complete-linkage method combined with Euclidean distance was used to construct clustering. (**C**) Correlation between m^6^A methylation regulators. Red and blue represent positive and negative correlation, respectively. (**D**) Mutation frequency of m^6^A methylation regulators in early-stage LUAD. The numbers and barplot on the right represent the mutation frequency of each m6A RNA methylation regulator and the proportion of each variant type, respectively.

In total, 22 m^6^A methylation regulators (2 erasers, 12 readers and 8 writers) were identified. Differential expression analysis revealed that m^6^A methylation regulators expression was abnormal in early-stage LUAD (Figure 1A, 1B). Moreover, 22 m^6^A regulators were basically positively correlated (Figure 1C). Among 381 samples, 85 samples had m^6^A methylation regulators mutations, and the mutation rate was 22.31% (Figure 1D). In early-stage LUAD samples, the ZC3H13 had the highest mutation frequency, followed by IGF2BP1, while WTAP did not show any mutation. These results imply that genetic and expression alteration landscapes of m^6^A methylation regulators are highly heterogeneous between normal and early-stage LUAD samples.

### Clustering based on m^6^A methylation regulators

**Figure 2:**
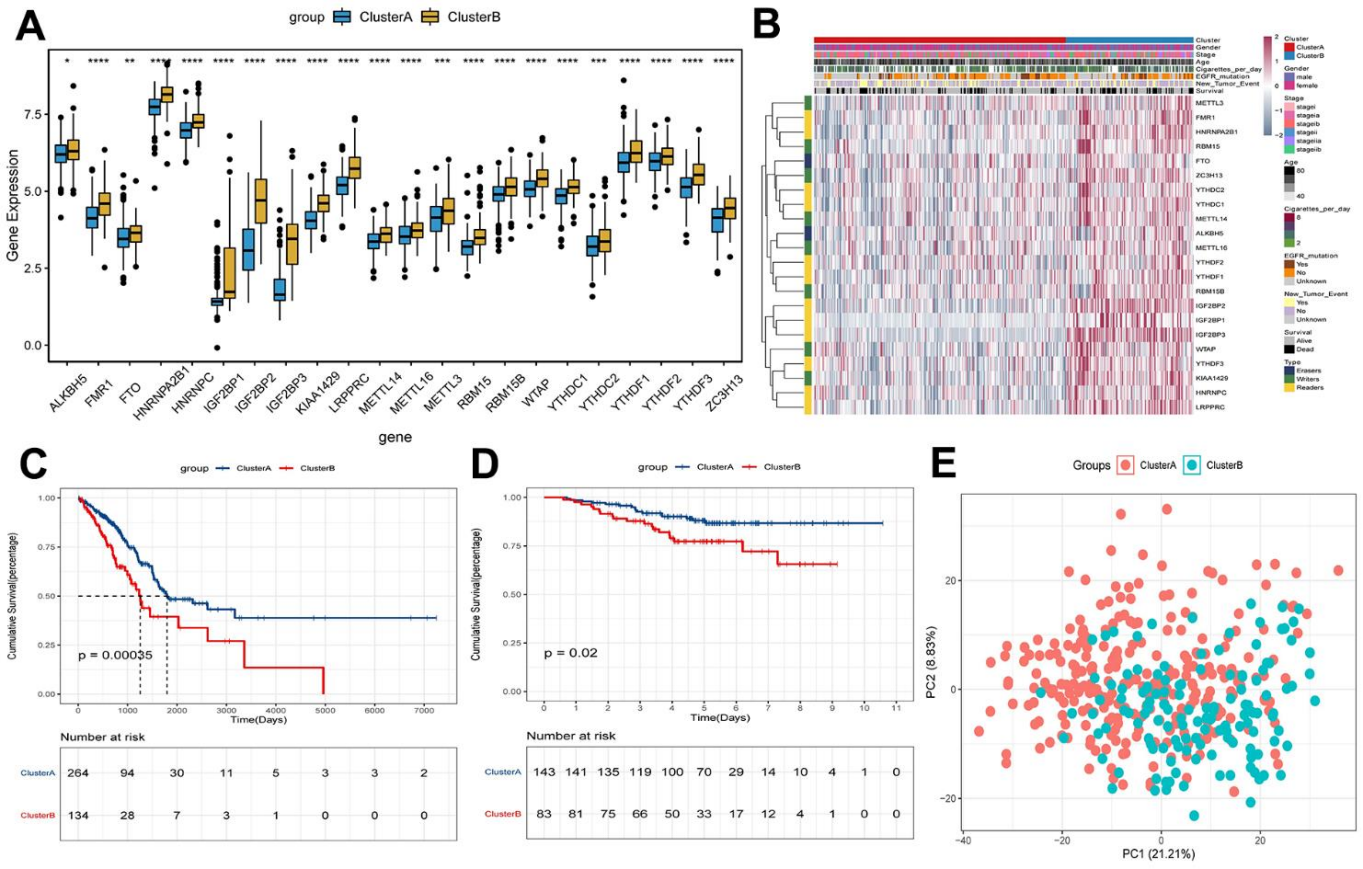
**Survival and expression analysis of m^6^A methylation regulators of early-stage LUAD in clusterA and clusterB subtypes.** (**A**) Expression boxplot of expression of regulatory factors in clusterA and clusterB subtypes in TCGA. *, P < 0.05; **, P < 0.01; ***, P < 0.001; ****, P < 0.0001. (**B**) Heatmap and clinicopathologic features of the clusterA and clusterB subtypes in TCGA. (**C**) Kaplan-Meier survival curves of clusterA and clusterB subtypes in TCGA; (**D**) Kaplan-Meier survival curves of clusterA and clusterB subtypes in GEO; (**E**) PCA was used to analyze gene expression patterns of clusterA and clusterB subtypes in TCGA.

In the process of increasing the number of clusters K from 2 to 5, when K = 2, there is a higher intra-group correlation and a lower inter-group correlation (Supplementary Figure 1A–1E). Similar results were verified in GEO (Supplementary Figure 1F–1J). Thus, patients in TCGA were divided into clusterA (264 early-stage LUAD samples) and clusterB (134 early-stage LUAD samples) according to K=2. Moreover, verification in GEO also found similar results (clusterA contains 143 early-stage LUAD samples; clusterB contains 83 early-stage LUAD samples). The results of differential expression analysis showed that the expression levels of m^6^A methylation regulators in clusterB were higher than that in clusterA (Figure 2A). Then, the clinicopathological features between clusterA and clusterB were compared. The results showed that clusterB mainly contained male patients with early-stage LUAD and was associated with higher age and smoking frequency (Figure 2B). KM analysis in TCGA (Figure 2C) and GEO (Figure 2D) indicated that the survival time of patients in clusterB was significantly shorter than that in clusterA. We also used the PCA method to further analyze the gene expression patterns of clusterA and clusterB. Gene expression profiles between clusterA and clusterB were well distinguished (Figure 2E). Our data showed that the cluster subtypes defined by the expression of the m^6^A regulators were closely related to the heterogeneity of early-stage LUAD patients.

### Characteristics of immune cell infiltration in TIME

**Figure 3:**
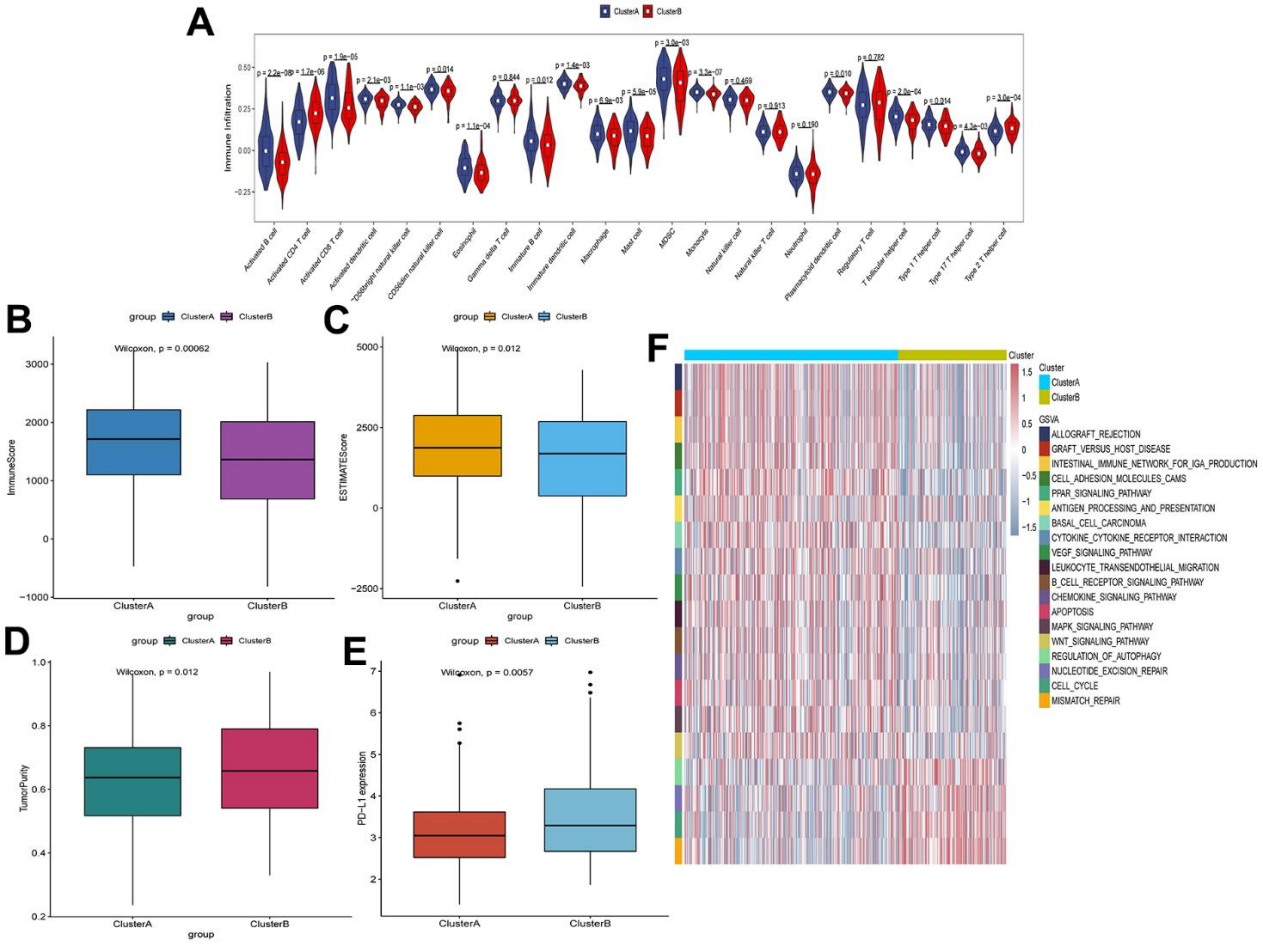
**Characteristics of cell infiltration in TIME of clusterA and clusterB subtypes in TCGA.** (**A**) The degree of immune cell infiltration in TIME of clusterA and clusterB subtypes; (**B**) Immunoscore in clusterA and clusterB subtypes; (**C**) ESTIMATEscore in clusterA and clusterB subtypes; (**D**) Tumor purity in clusterA and clusterB subtypes; (**E**) Expression level of PD-L1 in clusterA and clusterB subtypes; (**F**) GSVA enrichment analysis of clusterA and clusterB subtypes.

To investigate the degree of immune cell infiltration in TIME of clusterA and clusterB, we performed the ssGSEA analysis. The degree of immune cell (for example, Activated CD8 T cell and Immature B cell) infiltration in TIME was significantly reduced in clusterB (Figure 3A). Moreover, the immune and ESTIMATE scores were significantly lower in clusterB subtype than in clusterA subtype, and the tumor purity was significantly higher (Figure 3B–3D). We also detected the expression of PD-L1 in clusterA and clusterB. The expression of PD-L1 in clusterA was significantly higher than that in clusterB (Figure 3E). In addition, GSVA results showed that pathways (cell adhesion molecules, PPAR signaling pathway and B cell receptor signaling pathway) related to immunity and inhibition of tumor progression were significantly enriched in clusterA compared with clusterB (Figure 3F).

### Construction of m^6^A regulators prognostic model

**Figure 4:**
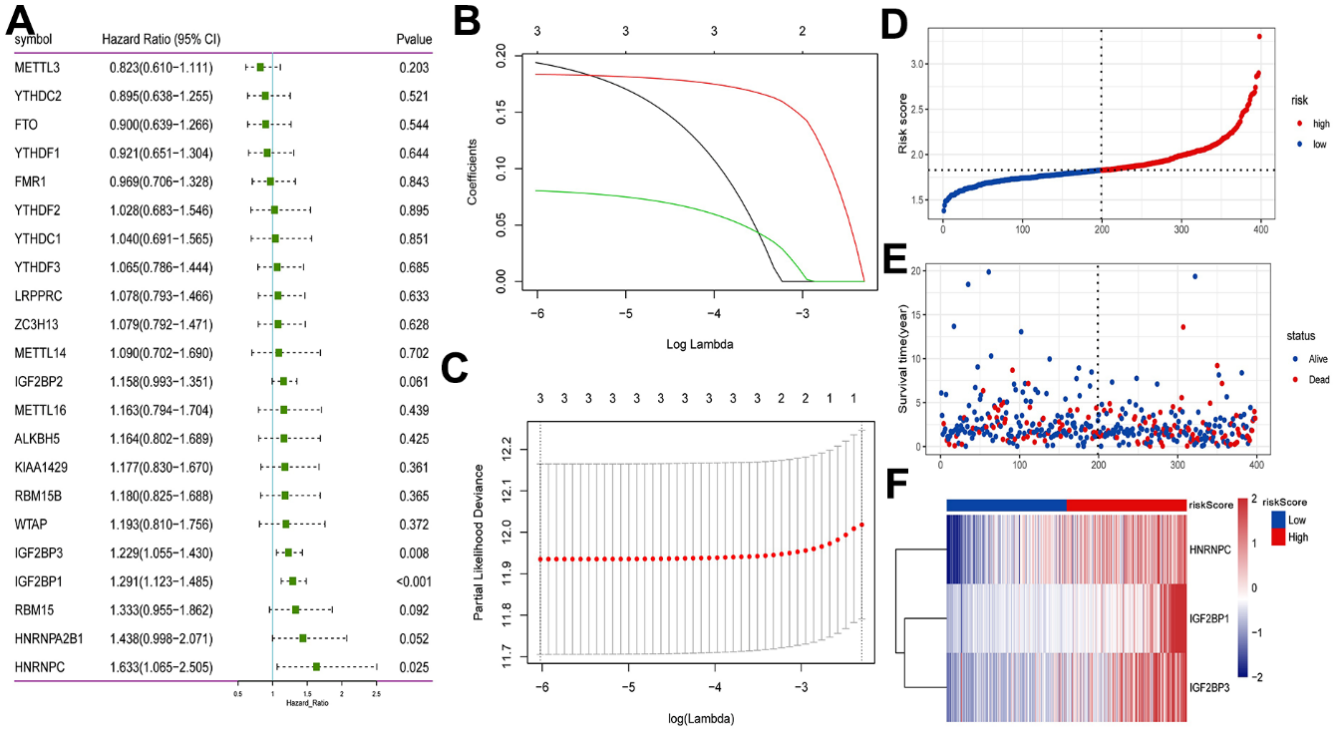
**Construction of prognostic model based on m^6^A methylation regulators in TCGA.** (**A**) Univariate Cox analysis screened out the m^6^A RNA methylation regulators that were significantly correlated with the prognosis of patients with early-stage LUAD. (**B**) The LASSO regression model was constructed; (**C**) the optimal value of the lambda penalty parameter was determined by 10-cross validation; (**D**) risk score distribution of early-stage LUAD. Red and blue represent high risk score and low risk score, respectively; (**E**) distribution of overall survival status of early-stage LUAD; (**F**) heat map of HNRNPC, IGF2BP1 and IGF2BP3. Red indicates above the reference channel. Blue indicates below the reference channel.

Univariate Cox analysis showed that 3 m^6^A regulators (HNRNPC, IGF2BP1 and IGF2BP3) were significantly associated with the prognosis of early-stage LUAD (Figure 4A). We performed LASSO-Cox regression analysis for the 3 characteristic genes. A prognostic model consisting of HNRNPC, IGF2BP1 and IGF2BP3 was constructed (Figure 4B, 4C). Risk Score = (0.1940*HNRNPC) + (0.1836*IGF2BP1) + (0.0805*IGF2BP3). Subsequently, the patients were categorized into high and low risk groups. The survival time of patients in high risk group was lower (Figure 4D, 4E). The results of heat map indicated that HNRNPC, IGF2BP1 and IGF2BP3 were lowly expressed in the lower risk group (Figure 4F). Drugs related to HNRNPC, IGF2BP1 and IGF2BP3 were screened based on DGIdb database (https://dgidb.org/). The results showed that only one drug DABIGATRAN related to HNRNPC was screened (Supplementary Figure 2).

**Figure 5:**
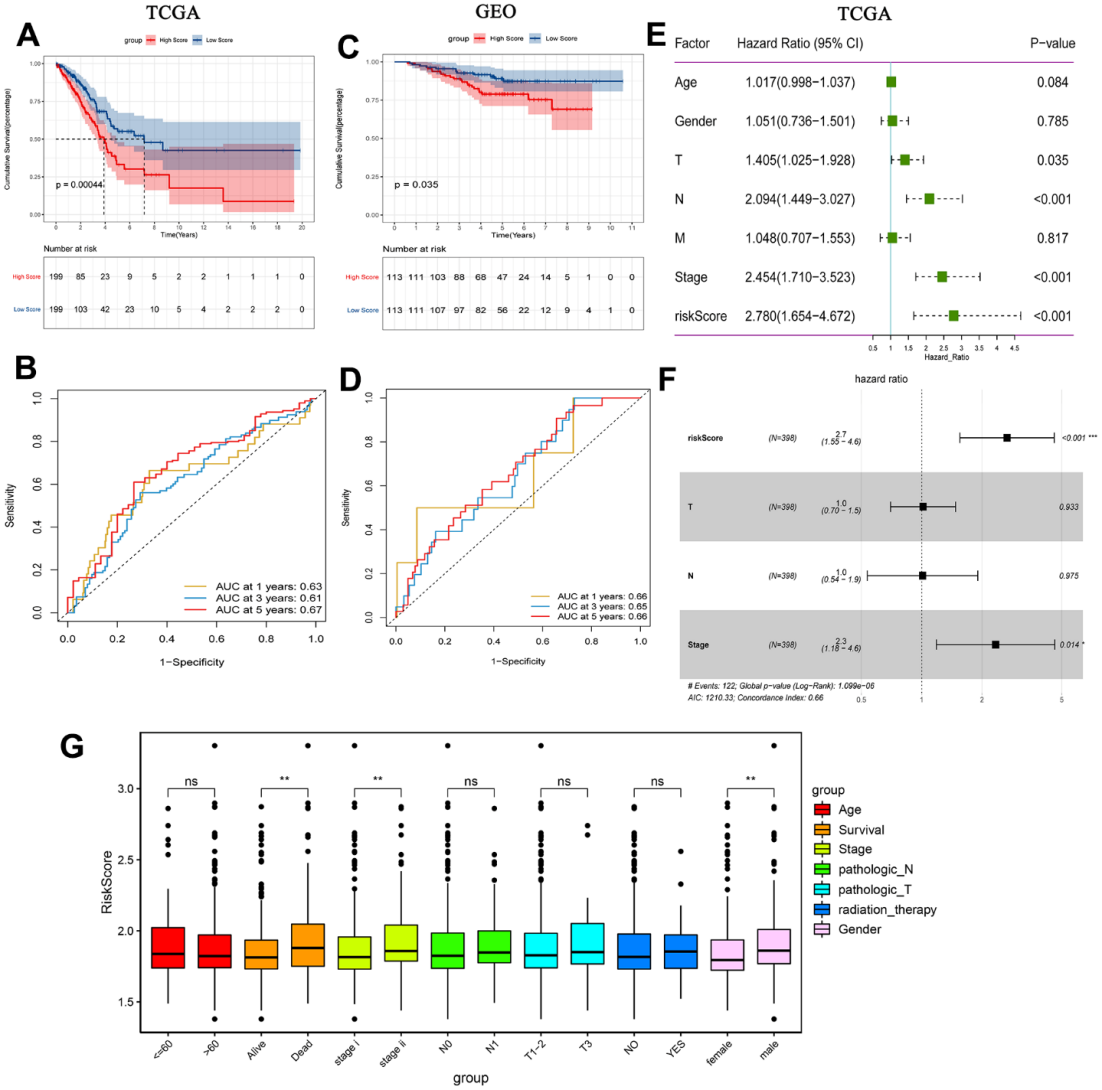
**Prognostic analysis of risk score.** (**A**) Kaplan-Meier survival analysis of early-stage LUAD in TCGA high and low risk score group; (**B**) Time-dependent ROC analysis measuring the predictive value of the risk score in TCGA; (**C**) Kaplan-Meier survival analysis of early-stage LUAD in GEO high and low risk score group; (**D**) Time-dependent ROC analysis measure the predictive value of the risk score in GEO; (**E**) Univariate Cox analysis confirmed that risk score was associated with overall survival in TCGA; (**F**) Multivariate Cox analysis found that risk score was an independent prognostic factor for early-stage LUAD in TCGA; (**G**) Correlation between risk score and clinical characteristics.

Additionally, patients in the high-risk group had a lower survival time, according to the KM analysis (Figure 5A). ROC curve analyses results showed that the AUC in 1, 3 and 5 years were all greater than 0.6 (Figure 5B). The results of KM analysis and ROC analysis in GEO (Figure 5C, 5D) and cBioPortal (https://www.cbioportal.org/) (Supplementary Figure 3A, 3B) databases are similar to those in TCGA. Decision curve analysis (DCA) was also performed based on TCGA. Prognostic indicator genes TP53 and NPM1 for LUAD and risk score were selected to perform DCA (Supplementary Figure 3C). The results of DCA showed that the predictive effect of risk score was better. Univariate analysis showed that T stage, N stage, TNM stage and risk score were correlated with overall survival of early-stage LUAD (Figure 5E). Moreover, multivariate analysis showed that TNM stage, risk score and overall survival were significantly correlated (Figure 5F). Additionally, risk score was significantly associated with survival, stage and gender (Figure 5G). In summary, the risk score may be an independent prognostic factor for early-stage LUAD.

### Effect of risk score on immune cell infiltration

**Figure 6:**
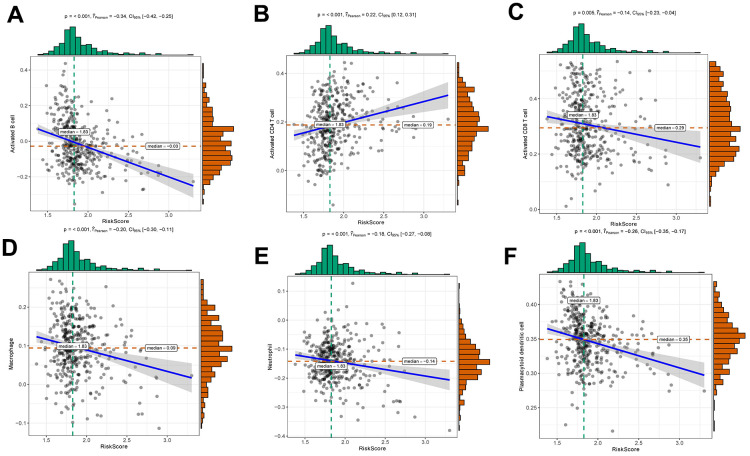
Relationships between risk score and activated B cell (**A**), activated CD4 T cell (**B**), activated CD8 T cell (**C**), macrophage (**D**), neutrophil (**E**) and plasmacytoid dendritic cell (**F**).

**Figure 7:**
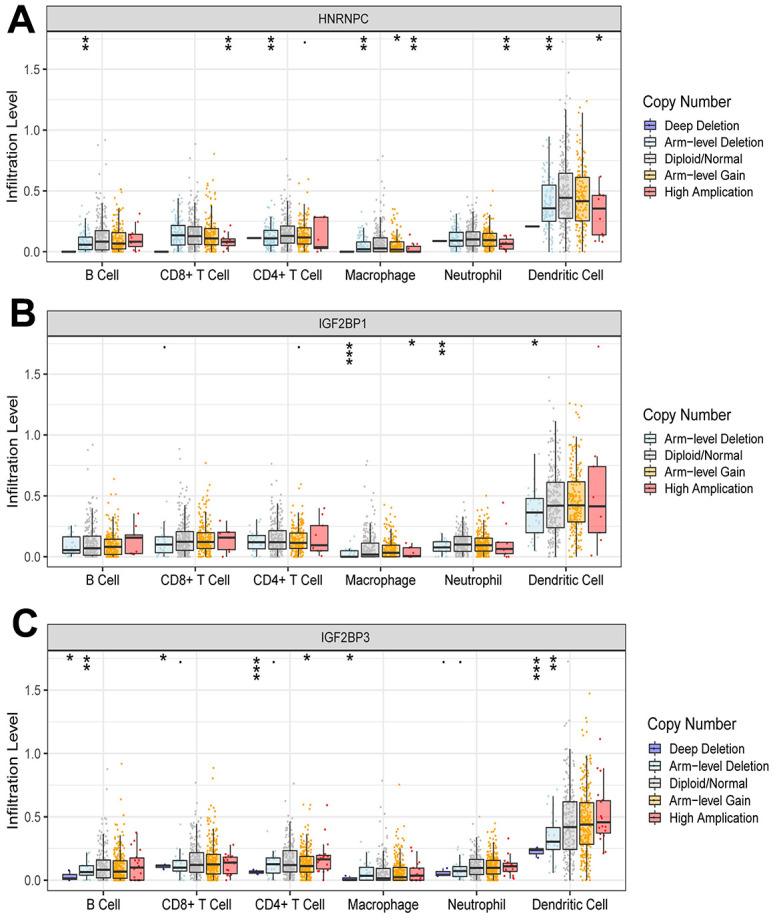
Effect of CNAs of HNRNPC (**A**), IGF2BP1 (**B**) and IGF2BP3 (**C**) on immune cell infiltration level. *, P < 0.05; **, P < 0.01; ***, P < 0.001.

The risk score was negatively correlated with the infiltration level of activated B cell, activated CD8 T cell, macrophage, neutrophil and plasmacytoid dendritic cell, and positively correlated with the infiltration level of activated CD4 T cell (Figure 6). This result confirmed that risk score based on HNRNPC, IGF2BP1 and IGF2BP3 was associated with the immune microenvironment of early-stage LUAD. Subsequently, we assessed the effect of CNAs of HNRNPC, IGF2BP1 and IGF2BP3 on the infiltration of immune cell. Somatic cell CNAs could affect the infiltration of immune cells (Figure 7).

### Expression validation of HNRNPC, IGF2BP1 and IGF2BP3 in clinical tissue samples through RT-PCR

**Figure 8:**
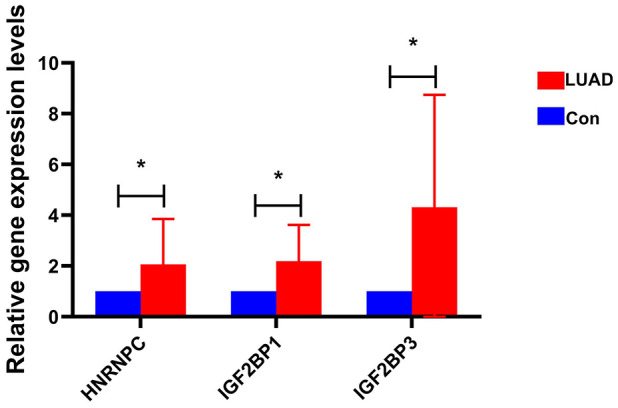
**The expression of HNRNPC, IGF2BP1 and IGF2BP3 in early-stage LUAD was verified by RT-PCR.** * represents P<0.05.

Primers of HNRNPC, IGF2BP1 and IGF2BP3 are shown in Supplementary Table 3. Expression levels of HNRNPC, IGF2BP1 and IGF2BP3 were significantly up-regulated in early-stage LUAD tissues compared with adjacent normal tissues (Figure 8). This result was consistent with bioinformatics analysis. This further indicates that HNRNPC, IGF2BP1 and IGF2BP3 may play important regulatory roles in the pathological mechanism of early-stage LUAD.

### Effect of HNRNPC on cell migration and invasion

**Figure 9:**
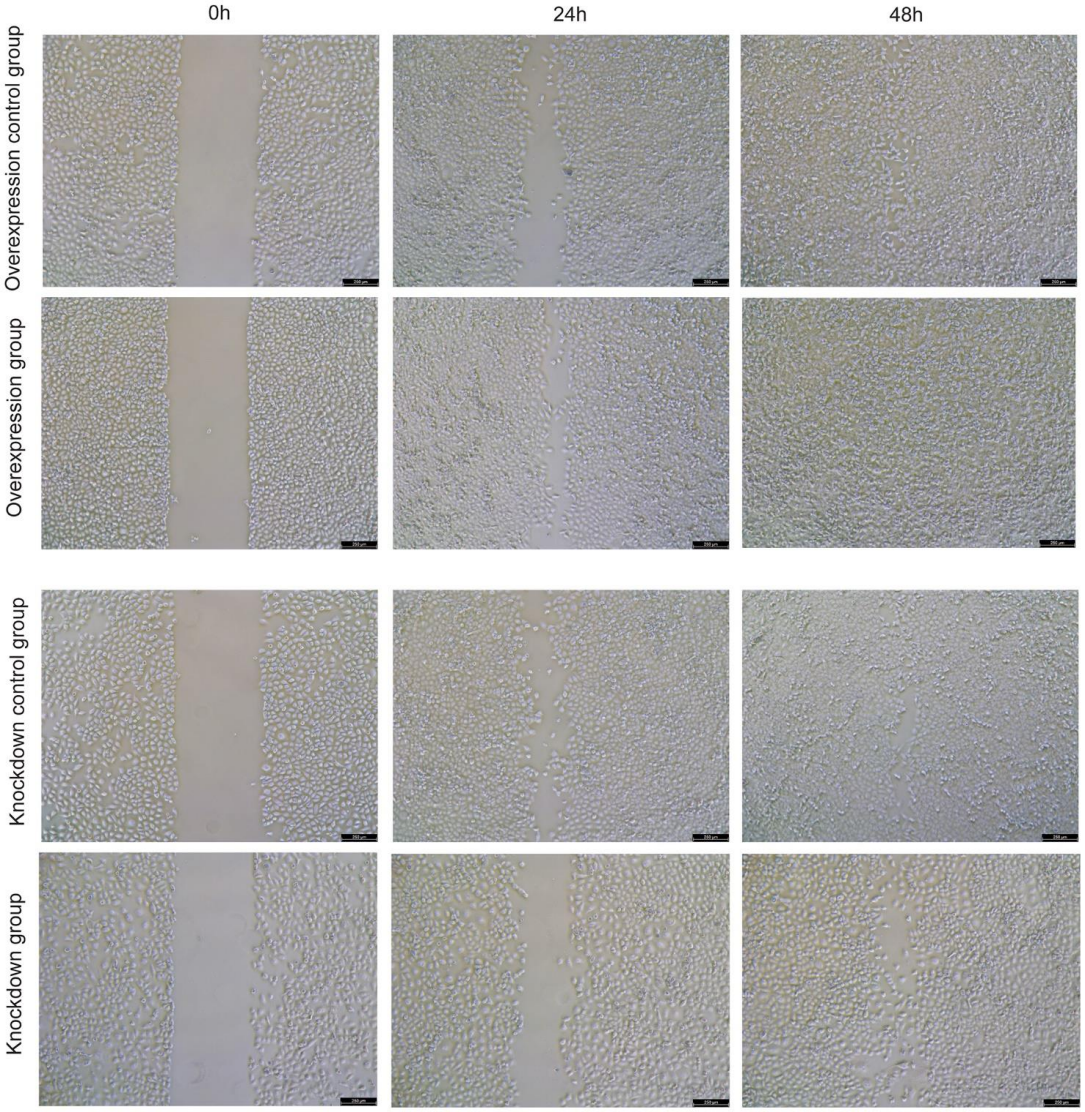
The effect of overexpression and knockdown of HNRNPC on the migration of NCI-H1299 cells was detected by cell scratch assay at 24 h and 48 h.

**Figure 10:**
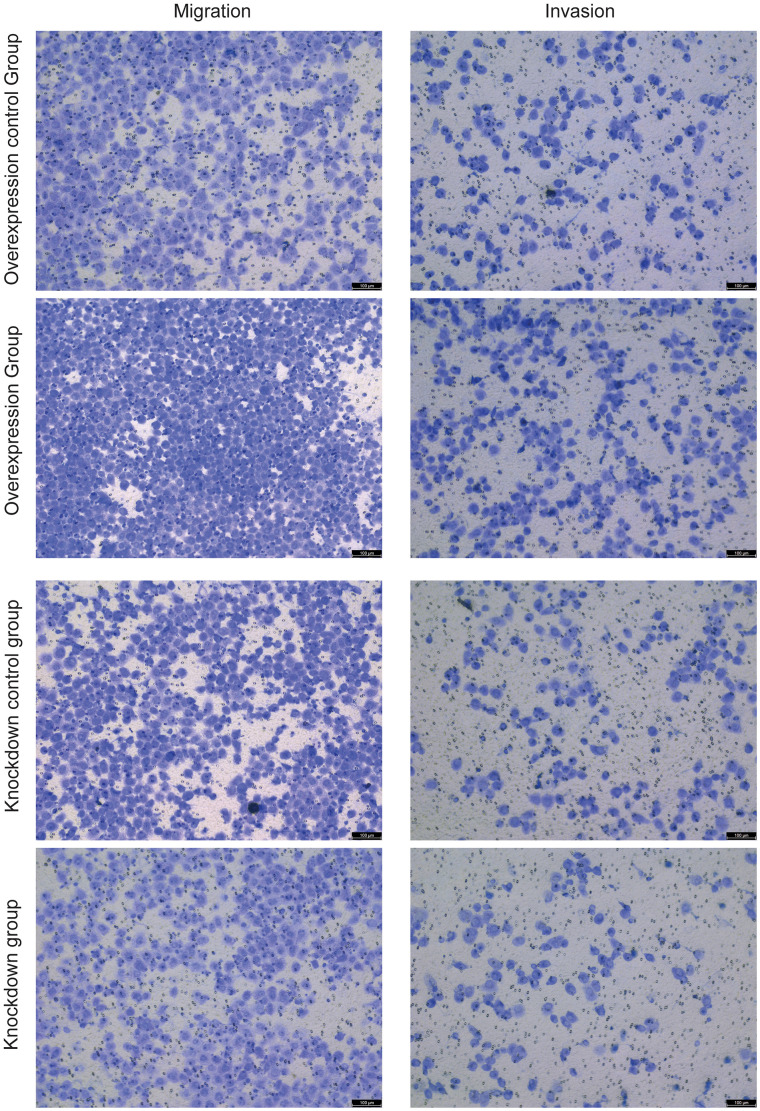
The effects of overexpression and knockdown of HNRNPC on the migration (left) and invasion (right) of NCI-H1299 cells were detected by cell scratch assay.

Cell scratch assay showed that overexpression of HNRNPC increased the migration ability of NCI-H1299 cells, while knockdown of HNRNPC decreased the migration ability (Figure 9 and Supplementary Figure 4A). Transwell assay also showed that overexpression of HNRNPC increased the migration ability of NCI-H1299 cells, while knockdown of HNRNPC decreased the migration ability (Figure 10 and Supplementary Figure 4B). In addition, Transwell assay also showed that overexpression of HNRNPC increased the invasion ability of NCI-H1299 cells, while knockdown of HNRNPC reduced the invasion ability (Figure 10 and Supplementary Figure 4C).

## DISCUSSION

With the progress of science and technology, more and more evidences indicate that m^6^A regulators play an important role in inflammation, immunity and anti-tumor [26, 27]. Exploring the role of m^6^A RNA regulators in TIME has become a hot research topic. LUAD is a common pulmonary malignant disease with a poor prognosis [28]. Therefore, exploring the effect of m^6^A regulators in the TIME of early-stage LUAD is beneficial for early management. In our study, we selected 22 m^6^A regulators for research in early-stage LUAD. Compared with normal controls, these m^6^A regulators showed obvious mutations and expression heterogeneity in early-stage LUAD.

Expression levels of m^6^A regulators in clusterB were higher than that in clusterA. KM analysis indicated that the survival time of patients in clusterB was significantly shorter. Notably, there are significant differences of TIME between clusterA and clusterB. In clusterB, infiltration degree of immune cells was significantly reduced. The higher the degree of infiltration of CD8 T cells and other immune cells in the TIME may indicate a better prognosis [29]. In addition, clusterB had lower immune and ESTIMATE scores, and higher tumor purity. Previous studies have indicated higher immune and ESTIMATE scores and lower tumor purity were associated with higher prognosis [30, 31]. We also studied the difference of PD-L1 in clusterA and clusterB. The expression of PD-L1 in clusterA was lower than that in clusterB. PD-1/PD-L1 can participate in regulating the immune escape of tumor cells [32]. The abnormally high expression of PD-L1 in TIME may be associated with activation of various carcinogenic signals [33]. These results indicate that the poor prognosis of clusterB may be related to immune cell infiltration and PD-L1 expression level.

The GSVA results showed that pathways related to immunity and inhibition of tumor progression were significantly enriched in clusterA compared with clusterB. Low cell adhesion molecules expression predicts poor prognosis of LUAD [34]. B cell receptor signaling pathway is an important pathway through which B cells recognize antigen and initiate immune response [35]. The up-regulation of PPAR signaling pathway can promote apoptosis of colorectal cancer cells and inhibit tumor progression [36]. PPAR signaling pathway also plays a vital regulatory role in LUAD [37]. Thus, we hypothesized that m^6^A regulators may play a regulatory role in the TIME of early-stage LUAD by regulating related signaling pathways.

A prognostic model was also constructed. The prognostic model consisted of 3 m^6^A regulators (HNRNPC, IGF2BP1 and IGF2BP3). It has been found that silencing heterogeneous nuclear ribonucleoprotein C (HNRNPC) can inhibit the proliferation and metastasis of prostate cancer cells. Furthermore, HNRNPC expression is negatively correlated with the levels of most immune cell infiltration in prostate cancer [38]. HNRNPC expression is lower in the high risk group of patients with lung squamous cell carcinoma and is more sensitive to immunotherapy and chemotherapy [39]. HNRNPC is significantly dysregulated and affects immune pathways and immune cell infiltration of endometriosis [40]. HNRNPC is also highly expressed in pancreatic adenocarcinoma and esophageal squamous cell carcinoma (ESCC), and can regulate the TIME [41, 42]. Herein, HNRNPC was highly expressed in early-stage LUAD tissues. Knockdown of HNRNPC inhibited the migration and invasion of NCI-H1299. Drug prediction results showed that DABIGATRAN was related to HNRNPC. Previous studies have shown that DABIGATRAN has some therapeutic efficacy in breast cancer, glioblastoma and pancreatic cancer [43, 44]. A case report indicates that DABIGATRAN was also involved in the treatment of a case of LUAD-associated hypercoagulability leading to venous limb gangrene [45]. The identification of DABIGATRAN provides new perspectives on the treatment of early-stage LUAD. Insulin like growth factor 2 mRNA binding protein (IGF2BP) 1 and IGF2BP3 belong to the same protein family. IGF2BP1 can regulate the invasion, migration, growth and proliferation of hepatocellular carcinoma (HCC) cells by long non-coding RNA LIN28B-AS1 regulation [46]. Increased expression of IGF2BP1 in endometrial cancer (EC) can promote cell proliferation and regulate tumor progression [47]. In ovarian cancer, expression of IGF2BP1 is negatively correlated with immune cell infiltration [48]. Inhibition of IGF2BP1 also inhibits the proliferation and migration of NSCLC cells [49]. In addition, high expression of IGF2BP1 in LUAD is related to poorer prognosis [50]. Down-regulation of IGF2BP3 in colon cancer can inhibit DNA replication and angiogenesis [51]. IGF2BP3 can also promote the occurrence of bladder cancer [52]. IGF2BP3 promotes the proliferation and migration of melanoma cells and is associated with the infiltration level of immune cell in the TIME [53]. In addition, IGF2BP3 is also related to the expression of immunomodulators and immune infiltration in NSCLC [54]. These findings suggest that HNRNPC, IGF2BP1 and IGF2BP3 play key regulatory roles in the development of tumors. It also implies that the potential functions of HNRNPC, IGF2BP1 and IGF2BP3 in early-stage LUAD are worthy of further study.

The relationship between immune cell infiltration and risk score was analyzed. Immune cell infiltration is associated with the prognosis of digestive system tumors and LUAD [55, 56]. Increased levels of CD8 T cell infiltration are associated with improved prognosis in NSCLC [57]. In this study, risk score was correlated with the level of immune cell infiltration. In addition, the somatic cell CNAs of m^6^A methylation regulators were found to influence the infiltration of immune cells. Thus, we hypothesized that m^6^A regulators may play a role in the prognosis of early-stage LUAD by regulating the TIME.

It is undeniable that our research has certain limitations. First of all, the data of this study were obtained through public databases, so a vast number of clinical sample data are needed for verification. Thus, a vast number of clinical sample data must be collected for further verification in the later stage. Second, our study only preliminarily revealed the correlation between m^6^A methylation regulators and prognosis and TIME of early-stage LUAD, and its specific molecular mechanism needs to be further studied.

## CONCLUSIONS

Expression analysis revealed that expression of m^6^A methylation regulators was abnormal in early-stage LUAD. Patients with early-stage LUAD were divided into two subtypes (clusterA and clusterB) based on the consensus clustering. The two subtypes have significant differences in TIME, prognosis, PD-L1 expression level and m^6^A regulators expression level. A prognostic model consisting of HNRNPC, IGF2BP1 and IGF2BP3 could be used to predict the prognosis of early-stage LUAD. Moreover, risk score was associated with infiltration level of immune cell in early-stage LUAD. Remarkably, overexpression of HNRNPC promotes the migration and invasion of NCI-H1299 cells, while knockdown HNRNPC inhibits the migration and invasion of NCI-H1299 cells.

## Supplementary Material

Supplementary Figures

Supplementary Tables
