# Supplementary material for: Consensus clustering and novel risk score model construction based on m6A methylation regulators to evaluate the prognosis and tumor immune microenvironment of early-stage lung adenocarcinoma
Source: Aging (Albany NY). 2024 Jul 5;16(14):11318–38. doi: 10.18632/aging.206004 (PMC11315395; doi:10.18632/aging.206004)
Supplement: Supplementary Figures [file aging-16-206004-s001.pdf]

SUPPLEMENTARY FIGURES

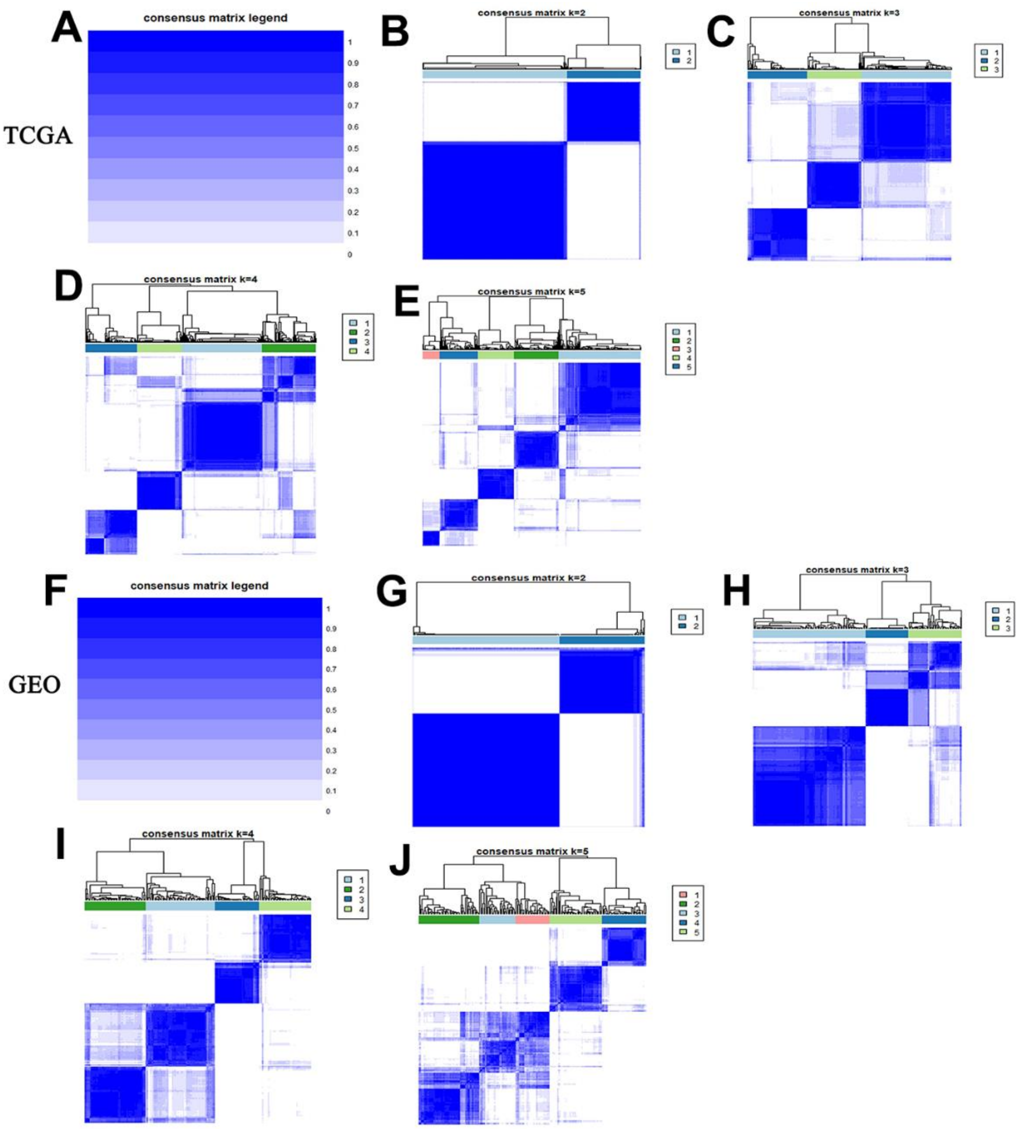

Supplementary Figure 1. Consensus clustering matrix analysis based on m^{6}A methylation regulators. (**A**) Color legend of consensus clustering matrix heat map in TCGA. The values of the consistency matrix from 0 (impossible to cluster together) to 1 (always cluster together) were represented by white to dark blue. (**B**) Consistent clustering matrix for k = 2 in TCGA. (**C**) Consistent clustering matrix for k = 3 in TCGA. (**D**) Consistent clustering matrix for k = 4 in TCGA. (**E**) Consistent clustering matrix for k = 5 in TCGA. (**F**) Color legend of consensus clustering matrix heat map in GEO. The values of the consistency matrix from 0 (impossible to cluster together) to 1 (always cluster together) were represented by white to dark blue. (**G**) Consistent clustering matrix for k = 2 in GEO. (**H**) Consistent clustering matrix for k = 3 in GEO. (**I**) Consistent clustering matrix for k = 4 in GEO. (**J**) Consistent clustering matrix for k = 5 in GEO. When taking different k values, the clustering effect was obviously different. The cleaner the clustering matrix, the better the effect.

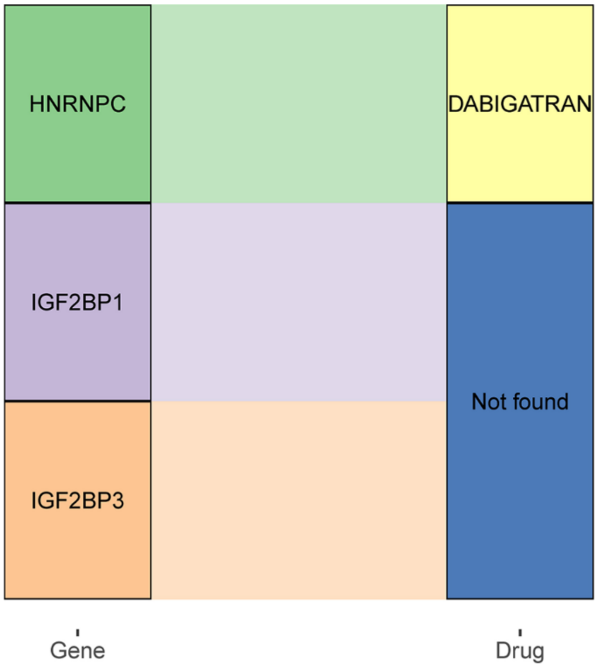

Supplementary Figure 2. Prediction of HNRNPC, IGF2BP1 and IGF2BP3 related drugs based on DGIdb database.

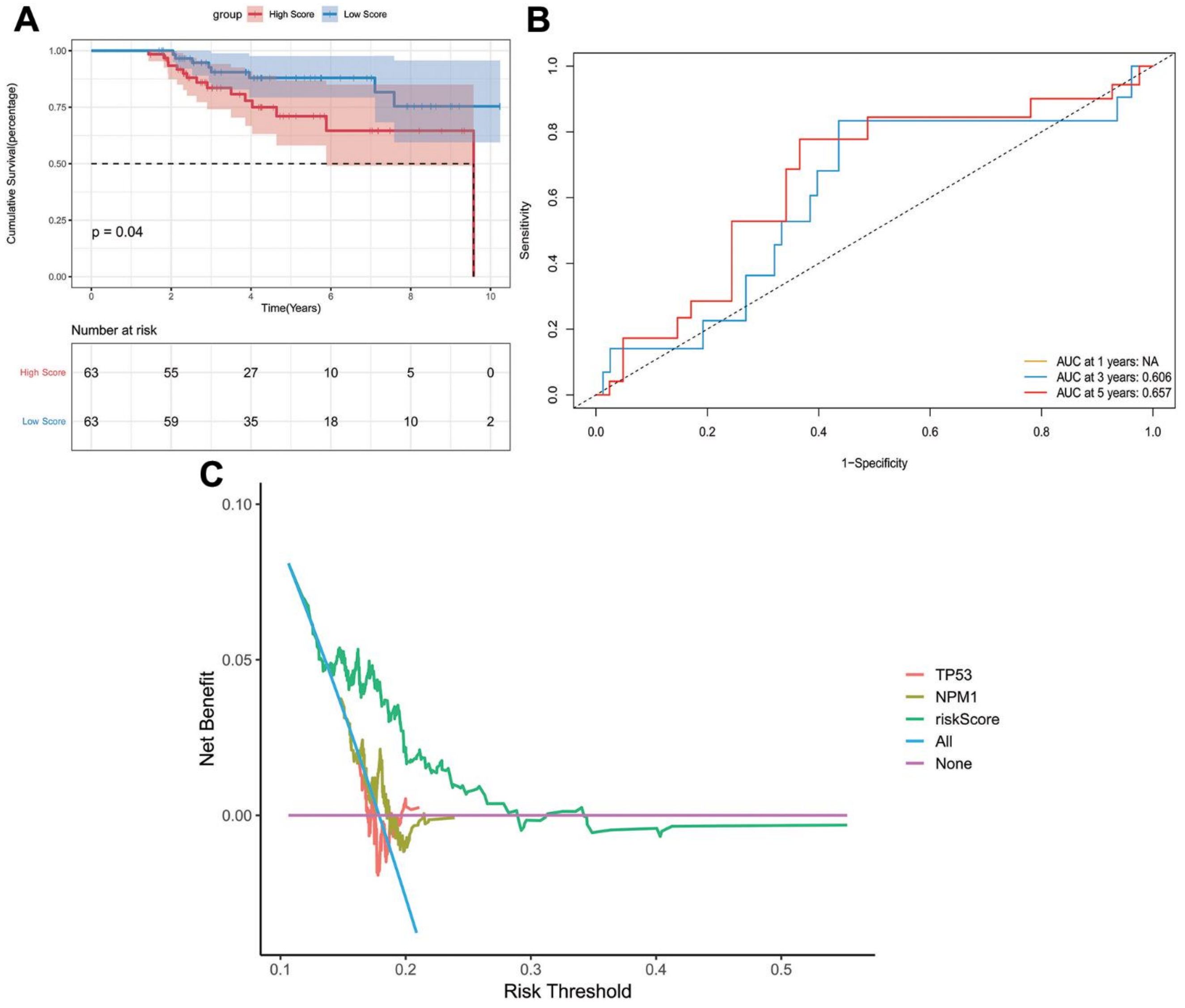

Supplementary Figure 3. Kaplan-Meier survival and time-dependent ROC analyses based on cBioPortal, and DCA based on TCGA. (**A**) Kaplan-Meier survival analysis of early-stage LUAD in cBioPortal high and low risk score group; (**B**) Time-dependent ROC analysis measure the predictive value of the risk score in cBioPortal; (**C**) Prognostic indicator genes TP53 and NPM1 for LUAD and risk score were selected to perform DCA based on TCGA.

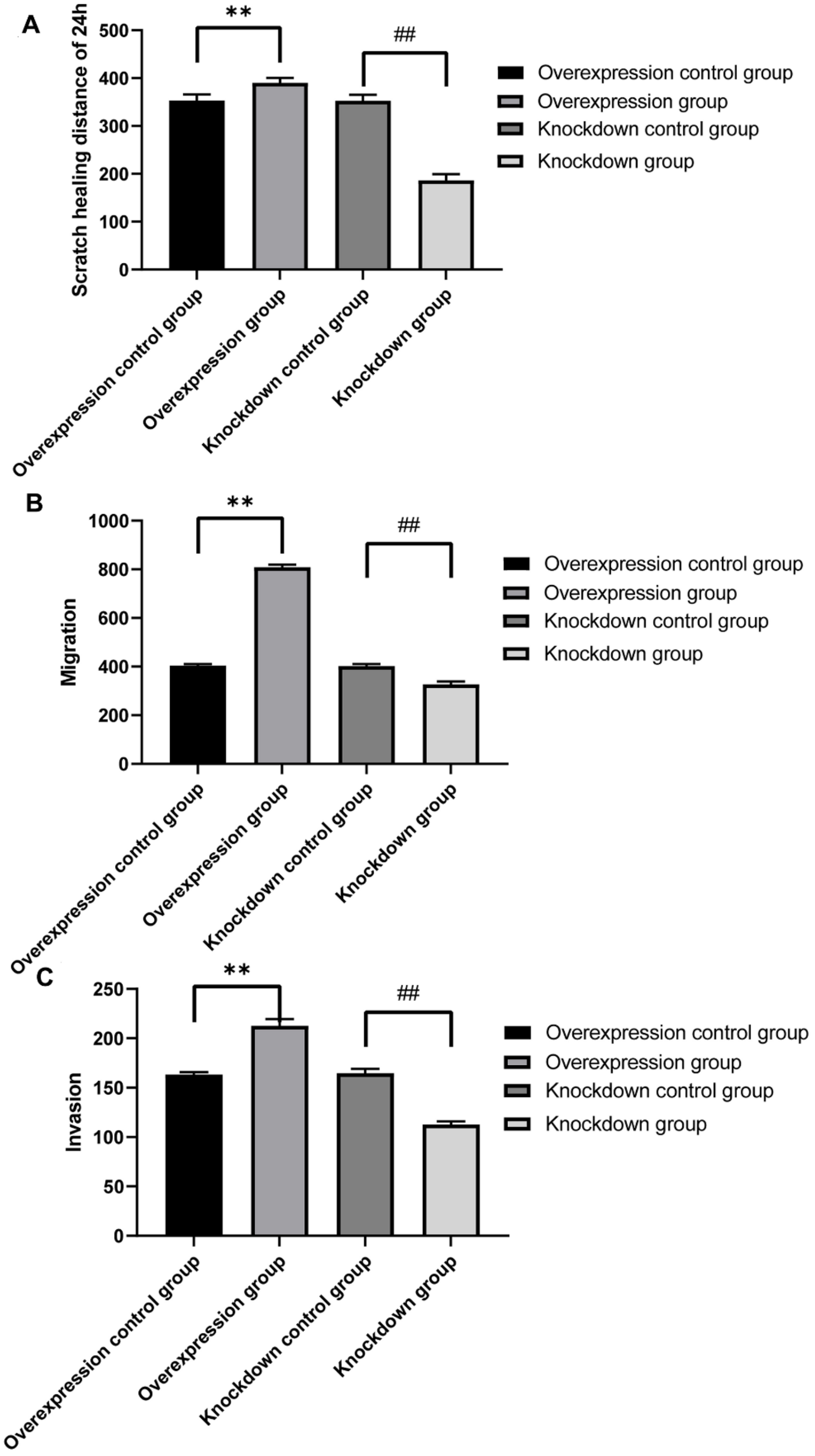

Supplementary Figure 4. One-way ANOVA was used to analyze the effect of HNRNPC on the migration and invasion of NCI-H1299 cells by SPSS17.0 software. (**A**) Statistical analysis of 24 h scratch healing distance of NCI-H1299 cells after HNRNPC overexpression and knockdown; (**B**) Statistical analysis of NCI-H1299 cells migration after HNRNPC overexpression and knockdown; (**C**) Statistical analysis of NCI-H1299 cells invasion after HNRNPC overexpression and knockdown. **P<0.01; ##P<0.01.
