# Supplementary material for: Consensus clustering and novel risk score model construction based on m6A methylation regulators to evaluate the prognosis and tumor immune microenvironment of early-stage lung adenocarcinoma
Source: Aging (Albany NY). 2024 Jul 5;16(14):11318–38. doi: 10.18632/aging.206004 (PMC11315395; doi:10.18632/aging.206004)
Supplement: Supplementary Tables [file aging-16-206004-s002.pdf]

SUPPLEMENTARY TABLES

Supplementary Table 1. Basic information of datasets.

Accession number /Source	Platform	Number of patients	Survival data
GEO: GSE31210	Affymetrix Human Genome U133 Plus 2.0 Array	Control: LUAD= 20:226	Overall Survival
TCGA: early-stage LUAD	Illumina RNAseq	Control: LUAD = 59:398	Overall Survival

GEO, Gene Expression Omnibus; TCGA, The Cancer Genome Atlas; LUAD, Lung adenocarcinoma.

Supplementary Table 2. Clinical information of patients in the RT-PCR.

Number	Sex	Age	Cancer metastasis	Pathological grade (stage I, II, III, IV)	Clinical stages (T stage, N stage, M stage)			Smoking history	Radiotherapy, chemotherapy and other treatment history	History of other malignancies	Family history of cancer
1	Female	50	No	IB	T2	N0	M0	No	No	No	No
2	Female	53	No	IA1	T1a	N0	M0	No	No	No	No
3	Male	43	No	IA1	T1a	N0	M0	Yes	No	No	No
4	Male	59	No	IA2	T1b	N0	M0	Yes	No	No	No
5	Male	57	No	IA1	T1a	N0	M0	Yes	No	No	No
6	Female	54	No	IA1	T1a	N0	M0	No	No	No	No
7	Female	47	No	IA1	T1a	N0	M0	No	No	No	No
8	Female	49	No	IB	T2a	N0	N0	No	No	No	No
9	Female	55	No	IA1	T1a	N0	N0	No	No	No	No
10	Male	66	No	IA2	T1b	N0	N0	No	No	No	No
11	Female	66	No	IA1	T1b	N0	N0	No	No	No	No
12	Female	52	No	IB	T2a	N0	N0	No	No	No	No
13	Male	52	No	IA2	T1b	N0	N0	Yes	No	No	No

Supplementary Table 3. Primer sequence in the RT-PCR.

Primer name	Primer sequence (5' to 3')
ACTB-F (internal reference)	5-CATGTACGTTGCTATCCAGGC-3
ACTB-R (internal reference)	5-CTCCTTAATGTCACGCACGAT-3
HNRNPC-F	5-GTTACCAACAAGACAGATCCTCG-3
HNRNPC-R	5-AGGCAAAGCCCTTATGAACAG-3
IGF2BP1-F	5-TGCAGTTTGGGTTGTGGACT-3
IGF2BP1-R	5-GATAGCTCCCAACTGCCTCC-3
IGF2BP3-F	5-CATCGAGGCGCTTTCAGGTA-3
IGF2BP3-R	5-CTCACAGCTCTCCACCACTC-3
